# Gestures make memories, but what kind? Patients with impaired procedural memory display disruptions in gesture production and comprehension

**DOI:** 10.3389/fnhum.2014.01054

**Published:** 2015-01-13

**Authors:** Nathaniel B. Klooster, Susan W. Cook, Ergun Y. Uc, Melissa C. Duff

**Affiliations:** ^1^Neuroscience Graduate Program, University of IowaIowa City, IA, USA; ^2^DeLTA Center, University of IowaIowa City, IA, USA; ^3^Department of Psychology, University of IowaIowa City, IA, USA; ^4^Department of Neurology, University of IowaIowa City, IA, USA; ^5^Neurology Service, Veterans Affairs Medical CenterIowa City, IA, USA; ^6^Department of Communication Sciences and Disorders, University of IowaIowa City, IA, USA

**Keywords:** hand gesture, procedural memory, declarative memory, Parkinson's disease, communication, learning, memory systems

## Abstract

Hand gesture, a ubiquitous feature of human interaction, facilitates communication. Gesture also facilitates new learning, benefiting speakers and listeners alike. Thus, gestures must impact cognition beyond simply supporting the expression of already-formed ideas. However, the cognitive and neural mechanisms supporting the effects of gesture on learning and memory are largely unknown. We hypothesized that gesture's ability to drive new learning is supported by procedural memory and that procedural memory deficits will disrupt gesture production and comprehension. We tested this proposal in patients with intact declarative memory, but impaired procedural memory as a consequence of Parkinson's disease (PD), and healthy comparison participants with intact declarative and procedural memory. In separate experiments, we manipulated the gestures participants saw and produced in a Tower of Hanoi (TOH) paradigm. In the first experiment, participants solved the task either on a physical board, requiring high arching movements to manipulate the discs from peg to peg, or on a computer, requiring only flat, sideways movements of the mouse. When explaining the task, healthy participants with intact procedural memory displayed evidence of their previous experience in their gestures, producing higher, more arching hand gestures after solving on a physical board, and smaller, flatter gestures after solving on a computer. In the second experiment, healthy participants who saw high arching hand gestures in an explanation prior to solving the task subsequently moved the mouse with significantly higher curvature than those who saw smaller, flatter gestures prior to solving the task. These patterns were absent in both gesture production and comprehension experiments in patients with procedural memory impairment. These findings suggest that the procedural memory system supports the ability of gesture to drive new learning.

## Introduction

People gesture when they speak. Hand gesture facilitates communication, helping transmit ideas from the speaker to the listener (Hostetter, 2011; Obermeier et al., 2012). Gesture provides information about the speaker's thoughts, and the information carried by gesture can either be unique to gesture or can reflect information also expressed in the accompanying speech. In this way, gesture provides a second informational stream during communication, both supplementing and complementing spoken language (Goldin-Meadow, 1999). Gesturing also benefits the speaker, increasing the fluency of the speech (Rauscher et al., 1996), reducing demand on working memory (Goldin-Meadow et al., 2001; Wagner et al., 2004; Ping and Goldin-Meadow, 2010; Cook et al., 2012), and facilitating lexical access (Rauscher et al., 1996; Beattie and Shovelton, 2000).

Gesturing goes beyond simply supporting the expression of already well-formed ideas, in that it also facilitates new learning and memory, and it does so for both speakers and listeners. Studies have shown that school children learn better when instruction contains both speech and gesture rather than speech alone (Perry et al., 1995; Valenzeno et al., 2003; Church et al., 2004; Ping and Goldin-Meadow, 2008, 2010). This learning is enhanced when gesture contains additional information not found in speech (Singer and Goldin-Meadow, 2005). It is also known that seeing gesture with speech facilitates later recall for the listener (Feyereisen, 2006; Kelly et al., 2009). Gesture also provides memory benefits for speakers, facilitating memory when produced at encoding (Cook et al., 2008, 2010; Hainselin et al., 2014), or at recall (Frick-Horbury and Guttentag, 1998; Stevanoni and Salmon, 2005).

Cook and Tanenhaus (2009) explored the role of gesture in learning and memory using a Tower of Hanoi (TOH) task to examine how the gestures listeners see influence their subsequent behavior, and how the gestures speakers produce reflect their previous experience. In their study, speakers first solved the TOH, either on a physical board that required them to manipulate weighted discs, or on a computer using a mouse to manipulate graphics of the discs. Speakers then explained the task to a partner (gesture production experiment). The authors found that the manner of speakers' previous motor experience in solving the task (real objects or computer) was reflected in their gestures during their explanations. Speakers who solved on a physical board used higher, more arching hand gestures reflecting their previous experience moving weighted discs, while speakers who solved with a mouse used flatter, sideways hand gestures similar to moving a mouse from side to side. Thus, the gestures speakers produced displayed memory of the manner of moving the discs when directly solving the task. The listening partners later solved the task themselves on a computer while their mouse trajectories were recorded (gesture comprehension experiment). In this study, listeners who saw high arching gestures and listeners who saw flatter gestures were equally good at solving the TOH. Nonetheless, the motor experience that was expressed in speakers' gestures influenced listeners' behavior. Listeners who saw higher gestures during the explanations used significantly higher, more arching mouse trajectories compared to listeners who saw flatter gestures. Thus, listeners learned how to move the discs from the gestures they saw and memory for this information was apparent in their subsequent mouse movements.

The work by Cook and Tanenhaus (2009) and others (Stevanoni and Salmon, 2005; Feyereisen, 2006; Hainselin et al., 2014) demonstrate the ubiquity and utility of gesture in driving new learning and memory. The cognitive and neural mechanisms that underlie this ability, however, are largely unknown. Empirical research from the cognitive neuroscience literature, and work with neuropsychological patients supports the notion that there are multiple (dissociable) learning and memory systems that rely on distinct neural structures (Scoville and Milner, 1957; Cohen and Squire, 1980; Squire, 1992; Bechara et al., 1995; Eichenbaum and Cohen, 2001; Henke, 2010). The two primary memory systems are declarative and non-declarative memory. Declarative memory, memory for facts about the world and the events of one's daily life, relies on the hippocampus and greater medial temporal lobes (Squire, 1992; Gabrieli, 1998; Eichenbaum and Cohen, 2001). Non-declarative memory, including procedural memory, priming, and classical conditioning, supports one's ability to acquire and perform skills and form habits through experience (Squire, 1992; Schacter et al., 1993; Gabrieli, 1998; Packard and Knowlton, 2002). The different types of non-declarative memory are supported by distinct brain regions and their interconnections, including the basal ganglia, cortex, and cerebellum (Packard and Knowlton, 2002).

Based on what is already known about gesture, memory systems, and learning, we hypothesize that the robust effect of gesture on driving new learning is supported by non-declarative memory. As a first attempt to test this hypothesis we examine the relationship between gesture and procedural memory. Our hypothesis, and the focus on procedural memory, is motivated by two observations. First, gestures are actions. The observed benefits in memory and learning come from the act of producing and experiencing gesture. This feature of gesture fits well with accounts of non-declarative memory resulting from direct experience, and with the role of procedural memory in support of motor, cognitive, and perceptual skill learning (Cohen and Squire, 1980; Poldrack et al., 1998; Knowlton and Moody, 2008). It also fits well with evidence that amnesic patients with impaired declarative memory but functional procedural memory benefit from performing actions during encoding—suggesting that performing actions engages procedural memory (Hainselin et al., 2014). Second, gesture's influence on learners is often implicit (i.e., not consciously accessible or verbalized) but influences behavior nonetheless (Goldin-Meadow et al., 1993; Garber et al., 1998). Although, consciousness alone does not reliably differentiate memory systems (see Hannula and Greene, 2012), there is agreement that the influence of procedural memory on behavior is outside of conscious awareness or introspection (Reber et al., 1996; Knowlton and Moody, 2008). Thus, the proposed relationship between gesture and procedural memory offers a parsimonious, and testable, account of gesture's ability to drive new learning.

Parkinson's disease (PD) is an often-used model of impaired procedural memory. In PD, the degeneration of midbrain dopaminergic neurons and the resulting striatal dysfunction leads to impaired performance on common laboratory measures of procedural memory (e.g., Rotary Pursuit or Serial Reaction Time tasks) while leaving performance on measures of hippocampal declarative memory (e.g., auditory verbal learning) relatively intact, particularly during the mild-moderate stages of the disease (Lees and Smith, 1983; Dubois and Pillon, 1996; Vakil and Herishanu-Naaman, 1998; Knowlton and Moody, 2008; although see Calabresi et al., 2013). Frontal and executive functions are a common cognitive impairment in PD as the disease progresses in severity (Zgaljardic et al., 2003). In addition, in contrast to their spared ability on many tests of declarative memory, patients with PD often show impairments on tasks that feature gradual learning of stimulus–response associations (Foerde and Shohamy, 2011). Although some studies do report impaired performance on declarative memory tasks, these deficits are often linked to frontal/executive deficits and not hippocampal pathology or the hippocampal declarative memory system (Zgaljardic et al., 2003). We should note that a few studies have shown hippocampal atrophy in PD, while other studies have not (for review see Calabresi et al., 2013). However, even with some reports of some degree of hippocampal atrophy, PD patients have disproportionate disruptions in procedural memory and any reported disruption in tasks that measure hippocampal declarative memory pale in contrast to the severe impairments in declarative memory associated with hippocampal amnesia (Knowlton et al., 1996; Squire and Zola, 1996; Gabrieli, 1998; Eichenbaum and Cohen, 2001).

Using a neuropsychological approach, we tested the specific hypothesis that patients with impaired procedural memory as a consequence of idiopathic PD would be impaired in gesture production and in gesture comprehension. A group of healthy non-brain damaged comparison participants (NCs), with intact declarative and procedural memory, and demographically matched to the patients, was also tested.

While there have not been previous studies of gesture's ability to drive learning and memory formation in PD, there have been investigations of gesture production in PD in communicative settings. Some of this work has shown that PD patients produce fewer gestures (Pitcairn et al., 1990; Duncan, 2008), produce gestures that are qualitatively less precise or inferior (Leiguarda et al., 2000; McNamara and Durso, 2003; Cleary et al., 2011; Bonivento et al., 2013), or produce gestures that are less expressive and that convey less intelligible information to the listener (Buck and Duffy, 1980). Less is known about gesture comprehension in PD and to, our knowledge, there are no studies that have examined the relationship between gesture production and comprehension and memory and learning in PD.

Here, our experimental paradigm followed the procedures of Cook and Tanenhaus (2009). We chose to extend this study for two reasons. First, many studies of the role of gesture in learning and memory use participants' verbal report to measure the effect of gesture on speakers and listeners (e.g., Cook et al., 2010; So et al., 2012). However, this approach limits the inferences that can be drawn about the nature of the memory system supporting gesture, as researchers are only measuring information in the declarative memory system (i.e., information accessible to conscious introspection and verbal report), and it is not clear that all information expressed in gesture emerges from declarative memory. The paradigm developed by Cook and Tanenhaus (2009) overcomes this limitation, by extending measurement of the effects of gesture on learning and memory into the perceptual-motor domain. Second, the TOH task used by Cook and Tanenhaus (2009) provides a platform for studying gesture that does not conflate gesture-associated learning and memory with task performance. Patients with impairments in procedural memory are likely to have difficulty in performing many tasks. In the Cook and Tanenhaus (2009) paradigm, participants do not need to solve the task normally or efficiently (i.e., using optimal strategies or a fewer number of moves) for the experimental effects to be evident in their behavior when solving the task and in their gestured explanations of the task. This is because the effects of gesture are seen in how participants move the discs when solving the task, and how participants gesture when explaining the task. Indeed, the effects in gesture production and comprehension in the work by Cook and Tanenhaus are observed in healthy participants even when there is considerable variability in task performance. That the gesture effects can be observed independently of task performance makes the TOH task ideal for studying gesture production and comprehension in patient populations who may vary considerably in task performance.

Given our hypothesis that gesture's power to drive learning and memory formation and its ability to reflect previous experience is supported by procedural memory, we predict that NCs will display the same patterns as observed in Cook and Tanenhaus (2009). When explaining the TOH (gesture production experiment), NC's should produce gestures that reflect their prior experience solving with a mouse or with physical discs. When solving the TOH after viewing an explanation (gesture comprehension experiment), NC's should show evidence of the gestures they saw in the explanation in their later mouse movements. Data from patients will implicate the role of procedural memory in this task. If patients with impaired procedural memory, but normal declarative memory, fail to replicate these patterns, this would provide strong evidence that gesture's effects on learning and memory rely on the procedural memory system. We predict that, in the gesture production experiment, after solving TOH with real objects and on the computer, PD patients will fail to display memory of their previous experience in the gestures they produce. Furthermore, in the gesture comprehension experiment, it is predicted that PD patients, unlike NCs, will also fail to show evidence of the previously seen gestures in their subsequent computer solutions.

## Materials and methods

### Participants

#### Parkinson's disease participants with procedural memory impairment

Nine mild-moderate, non-demented patients with PD (Hoehn and Yahr stages of 1–3) were enrolled in the study (Tables 1, 2). Diagnosis of idiopathic PD was based on the UK Brain Bank Criteria (Hughes et al., 1992). To be included, PD participants had to display impaired procedural memory, defined as less than 30% improvement across testing blocks when performing the Rotary Pursuit task (described below, Heindel et al., 1989). The PD group had a mean age of 64.4 (± 6.6) years and completed 15.2 (± 2.3) years of education on average. All patients were being treated with dopaminergic agents, with a group daily levodopa equivalent dose (LED) mean of 488.75 (± 188.55) mg/day (Tomlinson et al., 2010). Patients were tested on their regular medication schedule to enable good mobility and allow them to comfortably participate in study procedures. PD participants had intact declarative memory (i.e., within two standard deviations of normative means), as measured by the Rey Auditory Verbal Learning Task and the Rey-Osterreith Complex Figures Task. Neuropsychological testing also confirmed that the PD patients had intact visual-perceptual (*M* = 30.56 on Complex Figure Test Copy, *M* = 24.44 on Judgment of Line Orientation test, *M* = 6.79 on Benton Visual Retention Test) and executive function abilities (*M* = 5.67 categories completed on Wisconsin Card Sorting, *M* = 40 on COWA). A neurologist (EYU) examined patients at the time of testing and administered the Unified PD Rating Scale examination. Informed consent was obtained from the PD participants at the time of testing in accordance with procedures from the University of Iowa's Institutional Review Board.

**Table 1:** **Demographic characteristics of the patients with Parkinson's disease**.

**Subject**	**Sex**	**Age**	**Hand**	**Years of Ed**.	**HY**	**UPDRS Ment**.	**UPDRS ADL**	**UPDRS Mot**.	**LED**
PD028	M	68	R	12	2.00	0	7	13.0	540
PD046	M	75	R	16	2.00	1	14	17.5	560
PD051	F	73	R	12	2.00	0	9	21.5	350
PD058	F	62	L	16	2.00	1	5	7.0	450
PD066	M	59	R	18	2.00	2	9	14.5	850
PD079	F	55	R	13	2.00	4	7	11.5	648.75
PD086	F	59	R	16	2.00	3	11	14.0	300
PD094	F	64	R	18	2.00	2	1	11.5	300
PD104	M	65	R	16	2.00	1	14	26.5	400
PD Mean		64.4		15.22	2.00	1.56	8.56	15.2	488.75
NCs Mean		63.1		16.3	–	–	–	–	–

*Abbreviations: M, Male; F, Female; R, right-hand dominance; L, left-hand dominance; Years of Ed., Total years of education completed by the subject; HY, Hoehn and Yahr Scale; UPDRS, Unified Parkinson's disease Rating Scale; Ment., Mentation; ADL, Activities of Daily Life; Mot., Motor; LED, Levodopa Equivalent Dose (mg/day); NCs, Non-brain damaged healthy comparison participants*.

**Table 2:** **Neuropsychological characteristics of the patients with Parkinson's disease**.

**Subject**	**MMSE**	**RAVLT5/DR**	**RCFT Copy**	**RCFT Recall**	**WCST Cat./P.E**.	**COWA**	**Lines**	**BVRT C**	**BVRT E**	**TRLA T**	**TRLA E**	**TRLB T**	**TRLB E**	**TMT B-A**
PD028	29	11/7	26.0	17.0	6/6	34	29	5	7	32.71	0	60.63	0	27.92
PD046	30	9/4	23.0	9.0	3/22	63	29	6	9	35.04	0	87.75	1	52.71
PD051	29	15/12	34.0	29.0	6/13	40	19	8	3	22.6	0	76.6	2	54
PD058	30	15/13	29.0	12.5	6/4	44	25	7	3	20.65	0	44.07	0	23.42
PD066	30	12/13	35.0	30.0	6/7	52	30	7	4	21.6	0	52.47	1	30.87
PD079	30	9/6	33.0	20.5	6/14	24	20	8	2	22.45	0	54.03	0	31.58
PD086	30	11/9	35.0	33.0	6/5	48	18	8	2	30.95	0	80.77	0	49.82
PD094	29	10/11	36.0	15.0	6/8	29	25	8	3	39.41	0	63.27	0	23.86
PD104	29	11/11	24.0	10.5	6/9	26	25	4	8	46.72	0	106.11	0	59.39
PD Mean	29.56	11.44/9.56	30.56	19.61	5.67/9.78	40	24.44	6.78	4.56	30.24	0	69.52	0.44	39.29
NCs Mean	–	11.9/9.8	29.8	14.1	–	–	–	–	–	–	–	–	–	–

*Abbreviations: MMSE, Mini-mental State Examination; RAVLT, Rey Auditory Verbal Learning Test; 5, fifth block; DR, Delayed recall; RCFT, Rey Complex Figures Test; WCST, Wisconsin Card Sorting Test; Cat., Categories Completed; P.E., Perseverative Errors; COWA, Controlled Oral Word Association test; Lines, Judgment of Lines Orientation test; BVRT C, Benton Visual Retention Test correct; BVRT E, Benton Visual Retention Test errors; TRLA T, Trail Making Test A time; TRLA E, Trail Making Test A errors; TRLB T, Trail Making Test B time; TRLB E, Trail Making Test B errors; TMT B – A, time difference between Trail Making Test B time and Trail Making Test A time; NCs, Non-brain damaged healthy comparison participants*.

#### Non-brain damaged healthy comparison participants (NCs)

Non-brain damaged healthy comparison participants (NCs) included 18 individuals who were free of neurological disease or injury, and were matched to the PD patients on age, sex, handedness, and level of education. NCs participated in either the production task (*N* = 10), or the comprehension task (*N* = 8). To be included, all NCs had to display intact performance on the Rotor Pursuit task defined as a 30% or greater improvement across testing blocks (Table 3). NCs performed significantly better on the Rotor Pursuit task than patients (*t* = 5.31, *p* < 0.001, Table 3). NCs had a mean age of 63.1 (± 7.9) years and had completed 16.3 (± 1.8) years of education on average. *T*-tests revealed no significant differences between PD and NC participants for age (*p* < 0.63), education (*p* < 0.24), or declarative memory performance (AVLT recall, *p* < 0.84. CFT recall, *p* < 0.14). Informed consent was obtained from NC participants at the time of testing in accordance with procedures from the University of Iowa's Institutional Review Board.

**Table 3:** **Rotor Pursuit performance**.

**Group**	**Average speed**	**RP 1**	**RP 2**	***t*-value**	***p***
PD	35 rpm	67.253	76.237	−0.67	0.512
NC	52 rpm	47.166	68.935	−4.065	0.0003
		**PD**	**NC**	***t*-value**	***p***
Improvement		0.134	0.462	−5.31	0.00002

*Abbreviations: RP1, mean total time on target in the first testing block, in seconds; RP2, mean total time on target in the second testing block, in seconds; rpm, revolutions per minute; Improvement, % improvement from the second testing block compared to the first*.

### Experimental procedures

#### Rotor pursuit task

We measured procedural memory with the Rotor Pursuit task. All participants completed the Rotor Pursuit task following Heindel's protocol, which involves keeping a hand-held stylus on top of a moving target as it rotates around a circle (Heindel et al., 1989). Learning is shown by improving time on target across testing blocks. Before the testing blocks begin, participants are first tested at various speeds (15, 30, 45, and 60 rpm) for 20 s each to determine their baseline, which is defined as the speed at which participants stay on target for approximately 25% of the time. After their baseline is determined, participants complete two blocks of eight 20-s trials separated in time by at least 30 min. Learning is shown by increasing time on target across testing blocks. Comparing performance relative to baseline, rather than absolute performance is used to normalize performance, prevent ceiling effects from masking learning, and to give participants with varying levels of initial performance an equal chance to improve with experience.

#### Tower of Hanoi

The TOH task requires participants to move discs among three different pegs. Discs begin arranged on the leftmost peg with the largest on the bottom to smallest on the top. Participants' goal is to move the stack to the rightmost peg, moving one disc at a time and never placing a larger disc on top of a smaller disc. Participants solved the four-disc version of the task both on a physical tower (production task) and on a computer (production and comprehension tasks). The physical tower was taken from the testing materials of the Delis-Kaplan Executive Function System (D-KEFS) (Shunk et al., 2006). The computer version ran on Matlab on a 13″ laptop with a screen resolution of 1280 × 800. The computer program tracked the beginning and end time of each move, the total number of moves, and the x- and y-coordinates of the mouse cursor. On the computer, the discs could be transferred among pegs horizontally, without dragging the disc over the top of the peg. TOH is often used as a measure of executive functioning abilities (Shunk et al., 2006). As the patient group displayed normal performance on tests of executive functioning, we did not expect differences between groups on the task and task performance was not a main variable of interest. The TOH was included simply as a task during which we could manipulate the gestures participants viewed and measure the gestures that they produced.

#### Production task

To test gesture production, participants were asked to explain their solution to the TOH after solving on either a physical tower (Physical Condition) or on the computer (Computer Condition). Explanations were video recorded so that the gestures participants produced could be measured and analyzed. Participants then completed neuropsychological and experimental testing (for a mean of 88 min, ranging from 50 to 133 min), before solving the TOH task again under the other condition and again explaining their solution to the experimenter. Thus, our design was within-subjects and order of conditions was counter-balanced across participants. Gesture trajectory during participants' explanations was compared across conditions. To do so, all gestures that represented transferring a disc from one peg to another were annotated. Videos were exported to still images at 10 frames per second and hand trajectory was determined by recording the screen coordinates of the knuckle of the gesturing hand in each frame.

#### Comprehension task

To test gesture comprehension, participants were shown one of two videos of someone explaining how to solve TOH. Videos were culled from naturally elicited explanations and were matched for the content of the spoken explanation. One video was 89 s long and featured 11 high, arching hand gestures during the explanation (Curved Condition). The other video was 130 s long and featured 18 flat, sideways gestures (Flat Condition) as the explainer described the steps necessary to solve the puzzle. The gestures in the high arching condition were much more curved than those in the flat condition (In a linear mixed model using a quadratic function with video as a fixed effect, and random by video effects of intercept, *x*-value, and the x-quadratic term, there was a significant effect of video on the x-quadratic term, with the estimated curvature of arched condition = −1864, estimated curvature of flat condition = −521, *t* = 5.75, *p* < 0.0001). After watching the video explanation, participants solved TOH twice on the computer and their mouse trajectories were recorded so that their movement trajectories could be analyzed. Their mouse movements were used as the measure of gesture comprehension. Participants first viewed others' gestures and then their mouse trajectories were analyzed to look for evidence of which type of gesture they had just seen. After a period of neuropsychological and experimental testing (for a mean of 50 min, ranging from 33 to 70 min), participants watched the other video and solved again. Order was counter-balanced across participants.

PD participants completed the comprehension task first, as described above. The same participants completed the production experiment 6 months later (mean delay = 25.7 weeks, range = 10–58 weeks). Separating the two experiments over a period of months attempted to prevent the PD participants' experience seeing video explanations of solutions to TOH and solving the task in the comprehension experiment from affecting their behavior in the production task.

### Analysis of spoken explanations

After each task, participants explained their solutions of the TOH to the experimenter while being video recorded. Speech from these explanations was transcribed and each transcript was rated for content by two expert raters (researchers who were highly familiar with TOH explanations given by college undergraduates) blind to participant status and experimental hypothesis. The quality of each explanation was rated on a scale of 1 (poor) to 5 (excellent). The raters also guessed if the explanation came from a patient or a healthy participant, and guessed which experimental condition the participant had completed immediately prior to the explanation. Finally, the number of words used in the explanations, the number of gestures produced, and the gesture rate was calculated for each participant.

### Statistical methodology

#### Production

Our analysis followed Cook and Tanenhaus (2009). To analyze the trajectories of *hand motion* as recorded in screen coordinates, we used multilevel mixed effects regression models. Screen coordinates coded from participants' gestures representing individual moves back and forth across the board were transformed onto a common X scale, with the movement of each gesture ranging from a minimum of 0 (leftmost position) to 1 (rightmost position). *Y*-values were also transformed so that the minimum *y*-value of each gesture was 0. Because participants gestured about disc movement with curved trajectories, we modeled the hand position using a quadratic function—we expect a negative quadratic term reflecting the observed downward curve of the movements produced. Our model predicted the y position of the hand from the quadratic of the x position, along with a linear x term and an intercept. To assess for differences in the gesture trajectory associated with condition, each of these terms also interacted with condition. We included random by participant effects of intercept, *x*-value, and the x-quadratic term to allow each subject to have an individualized overall movement trajectory. Thus, our model looked for deviations from each individual's average movement trajectory as a function of experimental condition.

#### Comprehension

We again used a multilevel mixed effect model to analyze the *mouse* trajectories recorded during participants' TOH solution. To allow for analysis across different starting and ending points, X-coordinates for each move were transformed to a standardized scale of 0–1. We again used a quadratic function to model the predicted curved trajectory with condition as a fixed effect, and we included random by participant effects of intercept, *x*-value, and the x-quadratic term. Like the production model, this allows each participant to have an individualized average mouse trajectory, and looks for deviations from that trajectory as a function of the experimental condition.

## Results

### TOH solutions

We first examined performance on the TOH. We included data from the first solution to each of the comprehension conditions and from the computer production task. We did not include data from the real objects production task as this was not videotaped. We did not expect differences in performance across groups or experimental conditions, and, indeed, the mean number of moves needed to complete the task was remarkably similar between groups (PD mean = 31.3, NC mean = 31.2, *t* = 0.023, *p* < 0.98). Thus, patients and comparisons were equally successful in solving the task.

### Analysis of spoken explanations

Consistent with their equal success solving the TOH, analysis of spoken explanations revealed no differences in language use. The groups did not differ in the quantity or quality of their verbal explanations and there was nothing in their word use that revealed the condition they had just solved or their status as a PD patient or NC. More specifically, there were no significant group differences in the total number of words spoken, (PD mean = 585.6, NC mean = 575.6, *t* = 0.10, *p* < 0.92) or in the expert quality ratings for PD explanations and NC explanations for the production task (PD mean = 3.2, NC mean = 2.9, *t* = 1.18, *p* < 0.24) or the comprehension task (PD mean = 2.4, NC mean = 2.1, *t* = 1.13, *p* < 0.27). Our two expert raters, who have listened to hundreds of descriptions of the TOH, were not able to accurately identify participant status (PD patient or NC participant) or condition from the written transcripts The raters were unable to identify participant status at rates different than chance (Production: 55% correct, *t* = 0.84, *p* < 0.40. Comprehension: 47% correct, *t* = −0.36, *p* < 0.72). Similarly, the raters could not identify the condition the participant had just completed before their explanation at a rate different than chance (52% correct for production task, *t* = −0.36, *p* < 0.72; 42% correct for comprehension, *t* = 0.90, *p* < 0.37).

### Production

**Figure 1:**
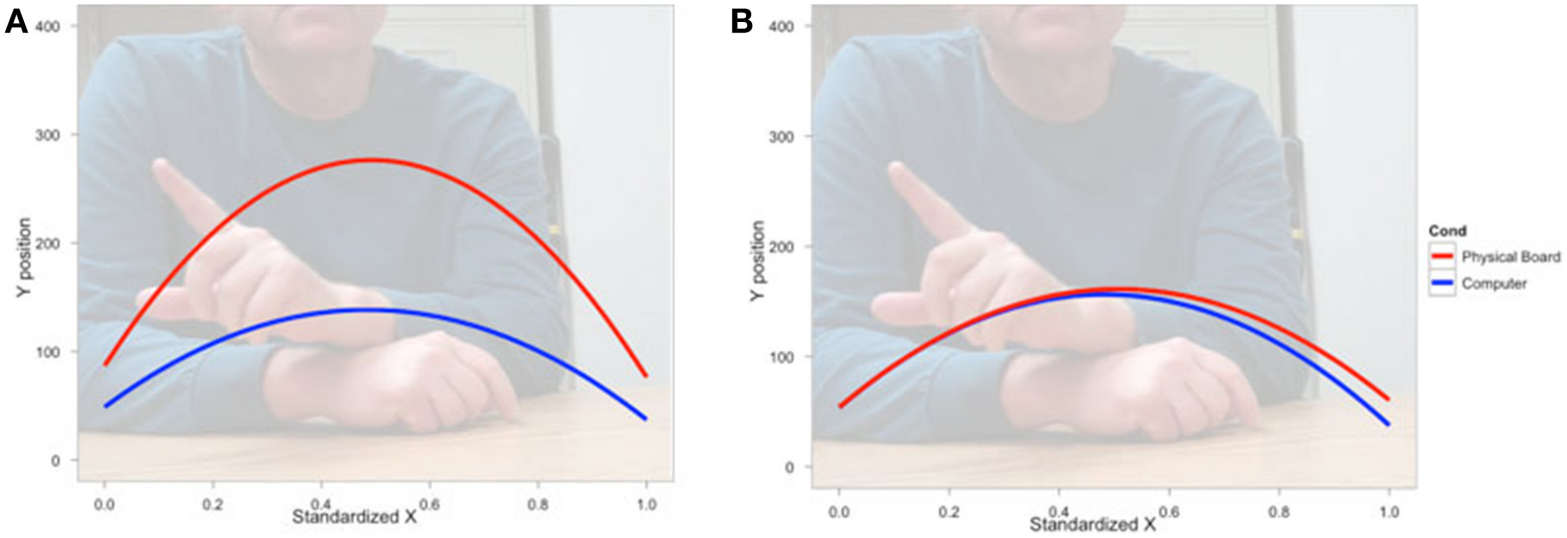
**Gesture production**. Predicted hand trajectories after solving the Tower of Hanoi with real objects or on a computer. **(A)** Healthy comparison participants produce gestures with significantly different trajectories after solving on the physical board compared with the gestures they produce after solving on a computer; **(B)** Participants with Parkinson's disease produce gestures that do not differ in trajectory across conditions.

We next asked how participants gestured with their hands during their explanations of the TOH (the gesture production experiment). One PD patient failed to gesture, so data from 8 PD patients and the 10 NCs were analyzed. Gestures were defined as hand movements depicting movement of a disc from one peg to another. Movements that did not depict disc motion were not coded. There were no differences between groups in number of gestures produced (PD mean = 65.13, NC mean = 57.10, *t* = 0.62, *p* < 0.55) or the gesture rate (gestures/word) during explanations (PD mean = 0.112, NC mean = 0.110, *t* = 0.33, *p* < 0.74). However, as can be seen in Figure 1 and Table 4, after solving on a physical board, NCs produced gestures with significantly more curvature (the coefficient of the quadratic term in our regression model) compared with the gestures they produced after solving on a computer (β_RealObjects_ = −397.88, *s.e*. = 38.18, *t* = −10.42, *p* < 0.001). NCs displayed reliably curved trajectories (β = −381.76, *s.e*. = 61.0, *t* = −6.25, *p* < 0.001). There was also a positive linear effect of × position (β = 370.0, *s.e*. = 57.41, *t* = 6.45, *p* < 0.001) that varied with condition (β_RealObjects_ = 398.8, *s.e*. = 38.3, *t* = 10.43, *p* < 0.001). The overall positive linear effect reflects an upward rightward trend in participants' hand position in the computer condition. There was no difference in curvature across conditions in the PD group (β_RealObjects_ = 26.5, *s.e*. = 30.5, *t* = 0.87, *p* < 0.384, see Table 5), although PD participants did have reliably curved trajectories (β = −442.6, *s.e*. = 127.1, *t* = −3.48, *p* < 0.01), as well as a positive linear effect of x position (β = 426.3, *s.e*. = 128.9, *t* = 3.31, *p* < 0.013) that did not vary with condition (β_RealObjects_ = −4.0, *s.e*. = 29.8, ns).

**Table 4:** **NC Production fixed effects**.

**Parameter**	**Estimate**	**Std. Error**	***t*-value**
Intercept	48.75	8.29	5.88^**^
X position	370.00	57.41	6.45^**^
Real objects	38.08	5.79	6.58^**^
X position × Real objects	398.82	38.24	10.43^**^
X position^2^	−381.76	61.05	−6.25^**^
X position^2^ × Real objects	−397.88	38.18	−10.42^**^

** *p < 0.01*.

**Table 5:** **PD Production fixed effects**.

**Parameter**	**Estimate**	**Std. Error**	***t*-value**
Intercept	53.669	12.045	4.46^**^
X position	426.278	128.872	3.31^**^
Real objects	0.358	4.479	0.08
X position × Real objects	−442.617	127.100	−3.48^**^
X position^2^	−4.004	29.842	−0.13
X position^2^ × Real objects	26.541	30.488	0.87

** *p < 0.01*.

### Comprehension

**Figure 2:**
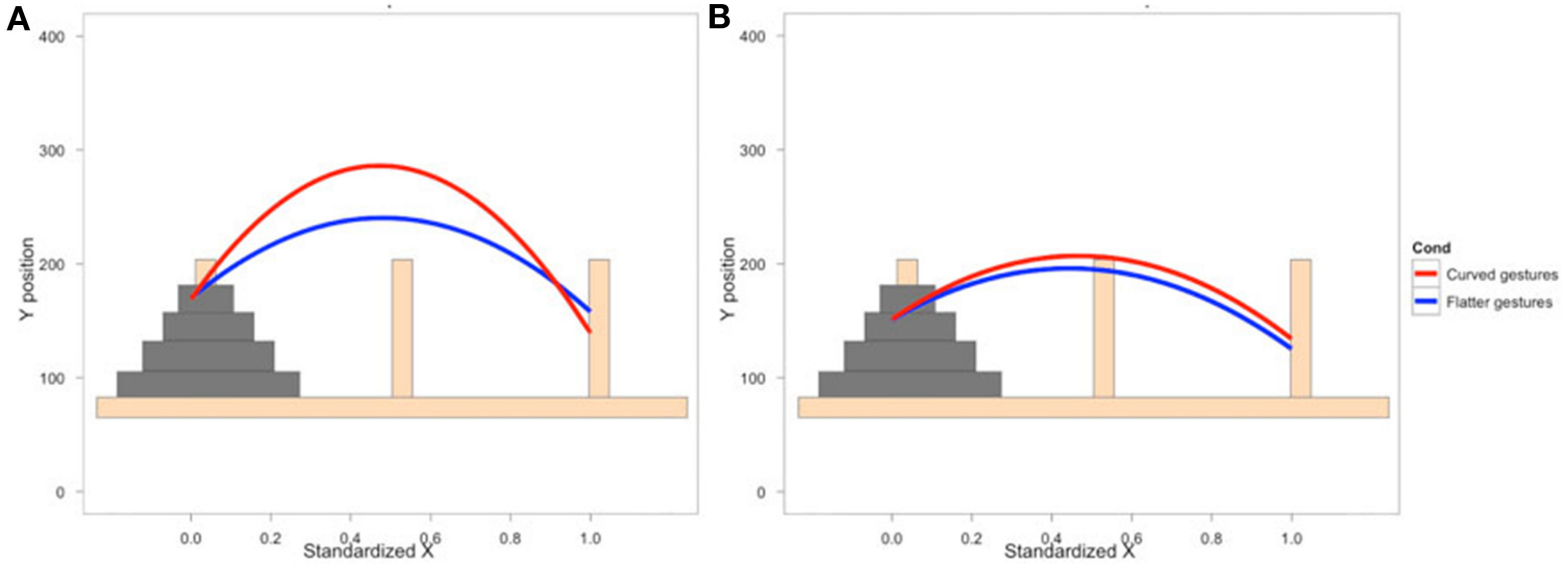
**Gesture comprehension**. Predicted mouse trajectories after viewing a video explanation of the Tower of Hanoi that included either curved hand gestures or flatter gestures. **(A)** Healthy comparison participants moved the mouse on the computerized task with significantly more curvature after seeing the video featuring high, arching gestures than after seeing the flat, sideways gesture video; **(B)** Participants with Parkinson's disease show no differences in curvature across conditions.

To examine how participants' behavior was influenced by the gestures they observed, we analyzed participants' mouse movements after viewing explanations with gestures of varying trajectories. Data from nine PD patients' and eight NCs' first three mouse moves were analyzed. We focused on the first three moves because we expected effects of condition to be most robust and observable for these moves. We expect the effect of the condition in the video to diminish with time as participants acquire their own experience moving the discs. Additionally, the first three moves involve placing discs on or near the bottom of the pegs (taking the first two discs off of the Tower and then typically moving the first disc onto the second disc) and thus the difference between curved and straight trajectories is larger. When the discs are being moved to higher positions, there is less of a difference between curved and straight trajectories, and when there are intervening posts and discs, trajectories are typically curved. As can be seen in Figure 2 and Table 6, NCs moved the mouse with significantly more curvature after seeing the video featuring high, arching gestures than after seeing the flat, sideways gesture video (β = −221.7, *s.e*. = 22.7, *t* = −9.78, *p* < 0.001). There was not a significant difference in curvature across conditions in the PD group (β = −26.9, *s.e*. = 14.1, *t* = −1.92, *p* < 0.06, see Table 7)—the trend for a difference in curvature in the PD group is an order of magnitude smaller than the effect observed in the comparison group. In both groups, trajectories were reliably curved (β_Parkinson's_ = −228.9, *s.e*. = 38.3, *t* = −5.97, *p* < 0.001; β_NC's_ = −306.0, *s.e*. = 98.4, *t* = −3.11, *p* < 0.017). There was also a positive linear effect of x position for both groups (β_Parkinson's_ = 203.3, *s.e*. = 42.4, *t* = 4.79, *p* < 0.001; β_NC's_ = 294.0, *s.e*. = 97.9, *t* = 3.0, *p* < 0.019) that varied with condition (β_Parkinson's_ = 35.2, *s.e*. = 14.9, *t* = 2.36, *p* < 0.018; β_NC's_ = 204.0, *s.e*. = 23.5, *t* = 8.7, *p* < 0.001).

**Table 6:** **NC Comprehension fixed effects**.

**Parameter**	**Estimate**	**Std. Error**	***t*-value**
Intercept	169.3	9.53	17.83^**^
X position	294.01	97.91	3.00^**^
Real objects	−1.20	4.02	−0.30
X position × Real objects	204.03	23.48	8.69^**^
X position^2^	−306.00	98.40	−3.11^**^
X position^2^ × Real objects	−221.67	22.68	−9.78^**^

** *p < 0.01*.

**Table 7:** **PD Comprehension fixed effects**.

**Parameter**	**Estimate**	**Std. Error**	***t*-value**
Intercept	150.852	6.801	22.18^***^
X position	203.262	42.399	4.79^**^
Real objects	0.522	2.948	0.18
X position × Real objects	−228.929	38.342	−5.97^**^
X position^2^	35.227	14.918	2.36^*^
X position^2^ × Real objects	−26.944	14.055	−1.92

* *p < 0.05*,

** *p < 0.01*,

*** *p < 0.0001*.

#### Deviations in vertical mouse movements

One possibility is that the observed disruptions in gesture comprehension in the PD patients can be explained by the presence of a motor impairment. That is, were PD patients influenced by the gestures they saw but this effect was masked by their motor impairment? The answer appears to be, no. If the patient's motor impairment were driving the failure to show different trajectories despite the different gestures seen prior to each solution, we would expect to see straight movements with little curvature across all of the moves necessary to solve the task, with PD patients failing to display deviations in their mouse movements and failing to produce high vertical deviations with the mouse. However, it was not the case that PD participants failed to produce these movements. In the PD group, eight of nine patients produced mouse movements with deviations greater than the NC average in the curved condition. Thus, we see variability in the mouse movements and heights of the movements in both NC and PD participants, and the PD patients are capable of making movements with trajectories resembling those produced by the NCs. An alternative possibility is that the movements of the PD patients were noisier, and that a true effect was obscured by noise in movement trajectories for the patients. However, we do not believe this to be the case as the variance in movement deviation was greater in the NC group, compared with the PD group (SD_PD_ = 58.1, SD_NC_ = 89.6), and this was true even when we only considered moves in the flat condition (SD_PD_ = 50.2, SD_NC_ = 86.0). That only the NCs (and not the PD patients) vary their movements consistently in response to the gestures they first see provides strong evidence that it is the impaired procedural memory in PD that explains their observed disruptions and not their motor impairment.

#### Non-criterion participants

Additional evidence for the relationship between gesture and procedural memory comes from the participants excluded from the main analyses for failing to meet the procedural memory criterion. Three PD patients scored too high on the procedural memory criterion and were therefore excluded from the primary analysis. These three participants did not differ from the included PD participants in their Hoehn and Yahr scores (*p* = 0.423) or in their UPDRS Motor sub-scores (*p* = 0.132) indicating that the severity of the disease and the extent of their motor impairment did not differ, but rather only their procedural memory proficiency differed from included PD participants. In the gesture comprehension experiment, these PD participants with intact procedural memory showed evidence of memory for the gestures they saw in the video explanations in their mouse movements. These three PD patients showed significantly higher curvature after seeing the high arching gesture video than the flat gesture video (β = −49.4, *s.e*. = 21.8, *t* = −2.3, *p* < 0.024). Surprisingly, there were also 12 NCs who failed to reach our procedural memory criterion and were excluded due to poor procedural memory performance. Not only did these participants not display the typical pattern of increased curvature after seeing the high arching gestures, they show an unexpected opposite pattern of results, with higher mouse movements after the smaller, flatter gesture video (β = 33.5, *s.e*. = 16.9, *t* = 1.99, *p* < 0.050).

We also have some preliminary evidence that a disruption in declarative memory does not affect gesture comprehension. While each included NC participant had intact procedural memory, and NCs as a group had intact declarative memory, three of the included NC participants in the comprehension experiment showed impaired declarative memory (i.e., two standard deviations below population means) on an individual basis. Interestingly, these three participants still display the same pattern of performance as the full NC group (β = −218.3, *s.e*. = 21.8, *t* = −9.99, *p* < 0.001), suggesting that a procedural memory deficit, but not a declarative memory impairment, disrupts learning from gesture.

Thus, the only differences between groups were the integrity of their procedural memory, the information in the gestures they produced, and the trajectory of their mouse movements after observing others' gestures.

## Discussion

We investigated gesture comprehension and production in a group of patients with intact declarative memory but impaired procedural memory, and a group of non-brain damaged comparison participants with normal declarative and procedural memory. We found that NCs' previous experience solving the TOH was apparent in the gestures they produced when explaining their solutions. Similarly, their behavior solving the TOH was influenced by the gestures they saw in a video explanation. These findings replicate previous results obtained with healthy college-aged participants in between-subjects versions of these tasks (Cook and Tanenhaus, 2009). These effects were absent in the patient group with impaired procedural memory, suggesting that intact procedural memory contributes to the capacity of gesture to drive new learning.

A difference between declarative and procedural memory is often described as the difference between *knowing how* vs. *knowing that* (e.g., Cohen and Squire, 1980). Procedural memory is described as memory for *how to* do something, as opposed to declarative memory, memory *that* something is the case. After seeing an explanation of an efficient solution to the TOH, healthy participants display memory of information in the gestures about *how* to manipulate the discs, using high arching movements after seeing high, arching gestures. They learned about the manner of how to manipulate the discs from the others' gestures. Similarly, memory of their previous experience solving TOH was apparent in their own explanations of how they solved the task. Their speech described the series of steps they used, while their gestures gave evidence of how they manipulated the discs, with higher, more curved gestures following their physical solutions and flatter, sideways gestures demonstrating how they manipulated the mouse on the computer solutions. These patterns were absent in the patient group with impaired procedural memory. These patients provided no information on how to move the discs in the gestures they produced as they explained the task. Likewise, after watching an explanation, they were not sensitive to information in the gestures they saw of how to manipulate the discs. This suggests procedural memory is necessary to learn and remember information experienced and expressed through hand gesture. As expected, performance on TOH did not differ between groups; these patients displayed intact executive functioning ability. Similarly, ratings of the groups' spoken explanations did not differ. Again this is not surprising given that both groups display intact declarative memory and the spoken explanations carried information from the declarative memory system. Procedural memory abilities did differ between groups and behavioral differences were seen in *how to* move the discs between groups.

To our knowledge, this study is the first to clearly link a specific memory system to hand gesture. Many studies have explored gesture's power to drive new learning and memory performance. This work has shown that for the speaker, gesturing at encoding facilitates later recall (Cook et al., 2008, 2010; Hainselin et al., 2014). Similarly, for the listener, seeing meaningful gestures along with speech facilitates later recall (Feyereisen, 2006). Gesturing at recall has also been shown to facilitate memory performance (Frick-Horbury and Guttentag, 1998; Stevanoni and Salmon, 2005). Despite the large literature detailing gesture's effects on learning and memory there has been little work linking gesture to memory systems and their neural substrates. Some work has shown that left hemisphere stroke patients show impairments in producing gesture on command in the absence of visual guidance (Roy et al., 1993). The authors interpreted this as a failure to perform these movements from memory and conclude that the left hemisphere supports memory for gesture performance. However, there has not been work linking gesture and memory at the level below an entire hemisphere, by connecting it to specific neural structures and specific memory systems. The findings here provide strong evidence that the procedural memory system supports at least some aspects of the learning afforded by comprehending and producing hand gesture.

While this study provides evidence that participants with intact procedural memory learned from the gestures that they see and produce, it is important to note that this learning did not help them solve the TOH. Indeed, this is an advantage of the TOH task; the influence of gesture on *how* participants solve the task can be manipulated without interfering with the ultimate solution. There is a large existing literature indicating that gestures facilitate learning. The goal of the current study was not to provide more evidence for this claim, but to begin to understand which learning systems available to learners are recruited by gesture, to begin to understand *how* gesture facilitates learning. Our proposal is that gesture may be an ideal candidate for conveying procedural information and bringing procedural knowledge into the learner's repertoire. The data from previous work (Cook and Tanenhaus, 2009) and the data presented here show that individuals learn content from the gestures they see, and the gestures they produce influence subsequent behavior. That is, even if that content does not help them solve the TOH task, they still acquired some information from the gestures they saw and experienced. Our proposal is that the ability to acquire information from gesture is supported by the procedural memory system. Our evidence for this proposal comes from the fact that individuals (with and without PD) differ in their ability to produce and comprehend the information conveyed in gestures as a function of their procedural memory status. Although their TOH performance was not affected by not having acquired this content, these data reveal a source of information that may not be available to these individuals in subsequent learning paradigms.

On the surface, the fact that the PD group in our study failed to display memory of their TOH solution in their subsequent gesture production may not be that surprising. This is a patient group suffering from a neurodegenerative disease known to cause motor impairments. It's important to note however that, like healthy participants, the PD participants in our study were capable of making the arching movements needed to move the physical discs up and off of a peg and over the top of a new peg, just as they were capable of moving a mouse flatly from side to side during the computer version of the task. In healthy older and younger adults, these different movement patterns are typically reflected in their later gestures. Although patients with PD were capable of these movements when solving the task, and recall that there were no differences in task performance as measured by number of moves, these patterns were absent in their later gestures, suggesting that it is the memory for these movements, rather than the physical actions themselves that are impaired.

The comprehension experiment provides compelling evidence that the deficits in the PD group are not limited to the motor system. Healthy participants with intact procedural memory learned from the gestures they saw and consistently varied their movements in response to the gestures they saw. The PD patients, although physically capable of the range of moments exhibited by the NCs, did not vary their movements as a function of their input. Non-criterion participants provide further evidence that procedural memory drives the gesture effects. NC participants excluded from the study due to poor procedural learning failed to show learning on the comprehension experiment, just like the PD group. Furthermore, three PD participants displayed excellent procedural memory and were excluded from analysis. These PD participants with intact procedural memory did learn from the gestures that they saw just like the NC group. These complimentary patterns provide strong evidence that procedural memory is necessary to learn from gesture, and impairments in procedural memory in the PD group underlies their failure to learn from gesture, not the motor complications from their neurodegenerative disease. These findings are consistent with the notion that procedural memory is not limited to motor learning but also encompasses cognitive and perceptual skill learning as well.

Other studies have shown that motor learning, that is, learning *how* to do something not just *what* to do, is enhanced through observation, much like our gesture comprehension experiment, where observing gesture provides information about how to move the discs. Motor learning can occur by simply observing the actions of others, without conscious awareness or conscious strategies (Mattar and Gribble, 2005). Action observation before training has been shown to enhance motor learning in healthy older adults (Celnik et al., 2006) and in chronic stroke patients, leading to improved motor functioning (Ertelt et al., 2007; Celnik et al., 2008). Indeed, much work has suggested a close link between action observation and the observer's motor system (Gallese et al., 1996; Rizzolatti et al., 1996) and it has been suggested that this can explain how hand gesture facilitates language comprehension (Glenberg and Gallese, 2012). Moreover, understanding others' gestures appears to rely on one's own motor system (Ping et al., 2014), perhaps providing a mechanism through which gesture influences later behavior as in Cook and Tanenhaus (2009). We have extended these findings by showing that healthy participants can learn from action observation—from the co-speech gestures they see during communication–while PD participants with impaired procedural memory cannot. This suggests that the integrity of the procedural memory system could be critical for the learning through observation reported both in our study and in the additional work described here.

While the focus of the current study was to examine a potential relationship between procedural memory and gesture, it is possible that other aspects of non-declarative memory also contribute to gesture's influences on learning. The most likely candidate would be priming. Priming influences behavior on a much quicker time course than procedural memory, which requires a gradual and extended learning process. Priming effects are usually observed with a single exposure to the prime stimulus whereas procedural memory tasks involve repeated trials before learning can be shown. For example, the Rotor Pursuit protocol we followed (Heindel et al., 1989) exposes the participant to 12 trials before comparing to a second set of eight trials to look for evidence of procedural learning. In a classic mirror reversed reading test of procedural memory (Cohen and Squire, 1980), participants were exposed to five words five times each before their learning was evaluated. Our participants produced or observed many gestures over the course of each experiment, consistent with the amount of practice needed for procedural learning. In the tasks we used, the most efficient solution to TOH is 15 moves, so participants had at least 15 experiences manipulating discs with their hands or with a mouse in the production task before they explained how to solve the task. In the comprehension task, participants observed 11 transfer gestures in the high, arching gesture condition and 18 transfer gestures in the flat, sideways gesture condition. Future work is needed to examine the time course of the learning driven by gesture to determine the potential role of priming, or an interaction between priming and procedural memory, in driving gesture's influence on behavior.

Given our proposal that gesture relies on non-declarative memory substrates to drive new learning, it may be possible to find a double dissociation–intact gesture production and comprehension in a population with intact procedural memory and impaired declarative memory. For example, patients with hippocampal damage and impaired declarative memory, but intact procedural memory would clarify the role of the declarative memory system in supporting gesture's impact on learning and memory. While such a demonstration awaits further study, we did find preliminary evidence for such an outcome in the three NC participants with impaired declarative memory and intact procedural learning who displayed the same learning effects from gesture as the full NC group. These preliminary findings suggest a limited role, if any, for declarative memory in the observed learning effects reported here.

The finding that gesture can exert its influence through non-declarative memory opens up new areas for investigation and new targets for rehabilitation in individuals with declarative memory impairment (e.g., Alzheimer's disease). Gesture is a large domain of behavior in which to look for signs of previous experience in memory impaired patients, particularly memories that may no longer be accessible to declarative memory or reportable through speech. Gesture is also a potential target for rehabilitation or intervention approaches. Gesture's proven role in reducing cognitive load particularly suggests that it receive further investigation with an eye out for how gesture can serve patients. Anything that can free up cognitive resources in patients struggling with impairments could potentially make a large impact. Indeed, one study has found that, in addition to enhancing normal speech, patients with focal brain injuries used gesture to compensate for impaired speaking abilities (Göksun et al., 2013). If gesture reduces cognitive load and reduces demands on working memory (Goldin-Meadow et al., 2001; Wagner et al., 2004; Ping and Goldin-Meadow, 2010; Cook et al., 2012), perhaps patients can be encouraged to gesture while they speak to benefit their communication. In addition, based on research showing that gesturing facilities memory when produced at encoding (Cook et al., 2010, 2008; Hainselin et al., 2014), or at recall (Frick-Horbury and Guttentag, 1998; Stevanoni and Salmon, 2005), encouraging patients with declarative memory impairments to gesture when speaking may be of benefit. Additional research is needed to explore these possibilities.

Future work is needed to investigate other types of gesture and their role in memory formation as well as how memory impairments in PD relate to the communicative function of gesture. The co-speech gestures investigated here are not the only type of gesture. Gesture is used by speakers communicating across contexts, and speakers use the manual modality in a variety of ways during communication. It makes great sense that different types of gestures (e.g., emblem gestures, beat gestures, interactive gestures, metaphoric gestures) could likely be supported by different memory systems and that the use of gestures (and the memory systems that support their use) would vary with task demands. While this study looked at gesture's role in learning and memory, other work is needed to see how gesture functions in communicative settings in patient groups. Some work has shown disrupted gesture production in PD in communicative settings (Buck and Duffy, 1980; Pitcairn et al., 1990; Leiguarda et al., 2000; McNamara and Durso, 2003; Duncan, 2008; Cleary et al., 2011; Bonivento et al., 2013). While the Buck and Duffy study, and the Bonivento et al., study provide no data on the disease severity of the included PD participants, with the exception of the Pitcairn study, all of these other examinations of PD and communicative gesture include patients with greater motor impairment and greater disease severity than the patients included in the current study. Interestingly, we found no difference in gesture rate between our PD group and the NCs. The current study included a PD group as a model of impaired procedural memory and the disease itself was not the primary variable of interest. Similarly, the current study investigated gesture's role in learning and memory, and not its role in communication. How procedural memory impairments, motor impairments, and other consequences of PD interact with and contribute to these communicative deficits is unknown, but the finding that gesture's influence on learning and memory relies on the procedural memory system provides new possibilities for investigation.

In investigating gesture comprehension and production, we found that in participants with intact procedural memory, evidence for previously experienced events was apparent in the gestures they produced when describing these events, and their behavior when solving a task was influenced by the gestures they saw in a video explanation of the task. These effects were absent in participants with impaired procedural memory, suggesting that intact procedural memory contributes to the capacity of gesture to drive new learning. Future work is needed to explore how other types of gesture relate to memory systems, and how memory deficits interact with communicative deficits in relation to gesture. The current study is the first to connect a large literature in psychology detailing gesture's power to driving learning and memory processes with a cognitive neuroscience literature on multiple memory systems and provides a starting point for future investigations in this area.

### Conflict of interest statement

The authors declare that the research was conducted in the absence of any commercial or financial relationships that could be construed as a potential conflict of interest.
